# FACTORS INFLUENCING PHYSICAL ACTIVITY AMONG COMMUNITY-DWELLING OLDER ADULTS IN BEIJING, CHINA: A PATH ANALYSIS

**DOI:** 10.1093/geroni/igae098.1177

**Published:** 2024-12-31

**Authors:** Ning Kang, Gong Chen, Dongmin Wang, Yue Wang, Min Liu

**Affiliations:** Peking University, Beijing, Beijing, China (People’s Republic); Peking University, Beijing, Beijing, China; Peking University, Beijing, Beijing, China (People’s Republic); Peking University, Beijing, Beijing, China (People’s Republic); Peking University, Beijing, Beijing, China (People’s Republic)

## Abstract

**Background:**

Previous research underscores the significance of physical activity in enhancing the health of older adults. This study seeks to delineate the pivotal determinants and model the pathways that influence the physical activity levels of older adults residing in community settings.

**Methods:**

Leveraging a cluster sampling strategy, this study surveyed older adults in Beijing, China, employing the International Physical Activity Questionnaire to assess physical activity. We gathered 770 valid responses (173 males and 594 females). The relationship between physical activity and various determinants was examined using ordered logistic regression, and a structural equation model was developed to elucidate the influencing pathways.

**Results:**

Findings reveal that a substantial portion of community-dwelling older adults exhibit low physical activity levels (40.00%), with moderate (28.7%) and high levels (31.30%) less prevalent. Direct influences on physical activity levels were found for exercise support (β=0.168), motivation (β=0.164), and knowledge (β=0.089). Exercise self-efficacy (β=0.21), support (β=0.152), and knowledge (β=0.092) indirectly affected motivation, with self-efficacy further mediated by support (β=0.081) and knowledge (β=-0.128), suggesting the nuanced role of exercise risk awareness. Exercise motivation, particularly achievement motivation, health motivation, and internal pressure motivation, was identified as a key factor affecting their physical activity levels.

**Conclusion:**

This research identifies exercise motivation and support as crucial to older adults’ physical activity. Contrary to expectations, increased self-efficacy does not directly correlate with higher activity levels, underscoring the complexity of factors influencing older adults’ engagement in physical activity.This study advise a community intervention enhancing exercise motivation through social support and health education.

